# Optimization of Ultrasound-Assisted Deep Eutectic Solvent Extraction and Mechanism Evaluation of Saponins from *Panax japonicus*

**DOI:** 10.3390/molecules31132200

**Published:** 2026-06-23

**Authors:** Jing Wang, Zhengwen Li, Xia Zeng, Miao Zheng, Minqian Wang, Qianlong Duan, Yong Jiang, Jia Li, Zhengyou He

**Affiliations:** Laboratory of Chinese Medicinal Resources & New Product Development, School of Pharmacy, Sichuan Industrial Institute of Antibiotics, Chengdu University, Chengdu 610106, China; 212024100700023@cdu.edu.cn (J.W.); lizhengwen@cdu.edu.cn (Z.L.);

**Keywords:** molecular dynamics, orthogonal experiment, choline chloride–urea, chikusetsusaponin IVa, ginsenoside Ro

## Abstract

This study investigated an efficient approach for extracting saponins from *Panax japonicus* using deep eutectic solvents (DES) coupled with ultrasound-assisted (UA) extraction, and compared its performance with the methanol extraction method. Twenty-six DES were screened, and choline chloride–urea was selected as the optimal solvent. The total extraction yield was evaluated based on the sum of the yields of chikusetsusaponin IVa (CS-IVa) and ginsenoside Ro (G-Ro). The extraction process was optimized using single-factor experiments combined with an orthogonal array design. Molecular dynamics (MD) simulation was applied to reveal the extraction mechanism at the molecular level. The results showed that the optimal conditions were as follows: a choline chloride-to-urea molar ratio of 1:3, a solid-to-liquid ratio of 1:50, a water content of 60%, an ultrasonic temperature of 40 °C, and an ultrasonic time of 60 min. Under these conditions, the total extraction yield of *Panax japonicus* saponins reached 7.4%, which was 13% higher than that obtained with the pharmacopeia methanol extraction method. MD simulation demonstrated that DES weakens intermolecular interactions among saponins through hydrogen bonds and van der Waals forces, promoting the dispersion of saponin aggregates and enabling efficient dissolution. Compared with CS-IVa, G-Ro displayed a more pronounced solvation effect, which was likely attributed to the difference in the number of polar sites in their molecular structures. The UA-DES extraction method established herein is green and efficient. It provides a practical reference for the industrial extraction of *Panax japonicus* saponins and a theoretical foundation for mechanistic studies on natural product extraction using DES.

## 1. Introduction

*Panax japonicus* C.A. Mey. (Araliaceae) is a highly valued traditional Chinese medicinal herb, for which the dried rhizome serves as the medicinal part [1,2]. According to traditional Chinese medicine theory, *Panax japonicus* possesses blood stasis-resolving, hemostatic, detumescent, analgesic, heat-clearing, lung-tonifying and replenishing activities [2]. Modern pharmacological studies have demonstrated that saponins are the principal bioactive constituents of *Panax japonicus* [3], and possess a wide range of pharmacological activities. Substantial preclinical evidence supports their neuroprotective effects, particularly in ameliorating age-related cognitive dysfunction [4], and they also exhibit significant potential in anti-inflammatory and antitumor effects [5,6]. Furthermore, *Panax japonicus* polysaccharides show considerable promise in antioxidant and immunomodulatory activities [7,8]. *Panax japonicus* requires at least five years of growth to meet harvesting standards, so its medicinal raw materials have long been heavily dependent on wild populations. Owing to overexploitation and habitat destruction, wild resources of *Panax japonicus* have decreased sharply, resulting in an increasingly prominent resource shortage [9]. Such resource scarcity has become a critical bottleneck restricting its pharmaceutical development and industrial application. Therefore, improving resource utilization efficiency and developing efficient extraction technologies for *Panax japonicus* are of great practical significance.

Currently, common methods for the extraction of saponins from *Panax japonicus* include ethanol extraction, water extraction, and enzyme-assisted extraction [10,11], all of which present considerable limitations. Water extraction is characterized by low efficiency and long extraction time, and the poor selectivity of water [12] leads to extracts with high levels of impurities [13]. Although ethanol is generally regarded as a relatively safe solvent, its organic residues and flammability, coupled with a low flash point and wide explosion range, pose safety risks in both laboratory operations and industrial-scale processes [14]. Enzyme-assisted extraction requires strict extraction conditions and high costs, rendering it unsuitable for large-scale production [15]. Therefore, the development of green and efficient extraction technologies is crucial for enhancing resource utilization of *Panax japonicus* and supporting the sustainable development of related industries.

Driven by the concept of green chemistry, deep eutectic solvents (DES) have become ideal alternatives to traditional organic solvents owing to their low toxicity, biodegradability, and environmental friendliness [16]. DES are eutectic mixtures formed by two or more safe and easily available components via hydrogen-bonding interactions [17]. In recent years, they have been widely employed in the extraction of various natural products, including saponins, polysaccharides, and alkaloids [18,19], demonstrating great promise. However, studies on DES-based extraction of *Panax japonicus* saponins remain scarce. Process optimization and related molecular interaction mechanisms for this extraction method are still in the early stages of exploration, and no mature technical system has been established yet.

In this study, targeting saponins from *Panax japonicus* as the active constituents, various DES formulations were first screened to identify the optimal system. Key parameters for DES-assisted extraction, including the molar ratio of hydrogen bond donor (HBD) to hydrogen bond acceptor (HBA), solid–liquid ratio, and water content, were further optimized. To explore the molecular basis, MD simulations were performed to reveal the solubilization and depolymerization effects of DES on *Panax japonicus* saponins, thus clarifying the inherent extraction mechanism. This study provides novel insights and technical support for the efficient utilization of *Panax japonicus* and other traditional Chinese herbal medicines.

## 2. Materials and Methods

### 2.1. Instruments and Materials

*Panax japonicus* was collected from an artificial forest in Baoshan City, Yunnan Province, China, and authenticated as genuine by Professor He Zhengyou from the Pharmacognosy Teaching and Research Section, Chengdu University (Chengdu, China). Reference standards, including Chikusetsusaponin IVa (CS-IVa) and ginsenoside Ro(G-Ro) (batch numbers: HS274W2, HS19115B2, Chenguang Biological Reference Co., Ltd., Handan, China), choline chloride (Shandong Keyuan Biochemical Co., Ltd., Heze, China, 99%), methanol, ethylene glycol, glycerol, urea, glucose (AR, Chengdu Kelong Chemicals Co., Ltd., Chengdu, China), malic acid (Changmao Biochemical Engineering Co., Ltd., Changzhou, China), and betaine (Shandong Aobo Biotechnology Co., Ltd., Liaocheng, China).

Analytical balance (Shanghai Shunyu Hengping Scientific Instrument Co., Ltd., Shanghai, China), Centrifuge (Beijing Jieke Technology Co., Ltd., Beijing, China), Multifunctional grinder (Dongguan Fangtai Electric Appliance Co., Ltd., Dongguan, China), Electric thermostatic blast drying oven (Shanghai Experimental Instrument General Factory, Shanghai, China), Ultrasonic cleaner (Kunshan Hechao Ultrasonic Instrument Co., Ltd., Kunshan, China), Agilent 1260 Infinity II high-performance liquid chromatography (HPLC) system (Agilent Technologies Co., Ltd., Santa Clara, CA, USA) equipped with OpenLAB CDS ChemStation Edition (version C.01.07 SR3, Agilent Technologies), Thermostatic water bath (Gongyi Yingyu High-Tech Instrument Factory, Gongyi, China).

### 2.2. Experimental Methods

#### 2.2.1. Material Pretreatment

The rhizomes of *Panax japonicus* were dried at 65 °C, ground, and sieved to 50–60 mesh. The powdered samples were further dried to constant weight in a drying oven at 50 °C, then bottled and sealed for future use.

#### 2.2.2. Determination Method

##### Preparation of the Test Solution

According to the Pharmacopeia of the People’s Republic of China [1,2], an appropriate amount of *Panax japonicus* powder was accurately weighed, dissolved in methanol, and diluted to volume. The solution was sonicated at 25 °C for 40 min to prepare the methanolic sample solution.

Separately, *Panax japonicus* powder and DES were mixed at a designated solid–liquid ratio in a 50 mL centrifuge tube. After sufficient mixing, ultrasonic extraction was conducted. The extract was centrifuged, diluted with methanol, and filtered to obtain the DES sample solution.

##### Reference Standard Solution Preparation

Accurately weighed appropriate amounts of CS-IVa and G-Ro reference standards, dissolved in methanol, diluted to volume, and filtered through a 0.22 μm microporous membrane for subsequent use.

##### Chromatographic Conditions

Column: NanoMicro C_18_ column (4.6 × 250 mm, 5 μm); flow rate 1.0 mL/min; injection volume 10 μL; column temperature 40 °C; detection wavelength 203 nm [20]. The mobile phase consisted of acetonitrile (A) and 0.1% phosphoric acid aqueous solution (B) under gradient elution as follows: 0–3 min, isocratic at 25% A; 3–11 min, linear gradient from 25% to 33% A; 11–35 min, isocratic at 33% A; 35–40 min, linear gradient from 33% to 37% A; 40–60 min, isocratic at 37% A.

#### 2.2.3. Method Validation

##### Linearity

Appropriate volumes of the reference standard solution under Section Reference Standard Solution Preparation were accurately pipetted and serially diluted. After filtration through a 0.22 μm microporous membrane, the solutions were injected and determined under the chromatographic conditions described in Section Chromatographic Conditions. Standard curves were constructed by plotting peak areas (Y) against the corresponding concentrations of the reference standards (X).

According to the Chinese Pharmacopeia [1], the limit of detection (LOD) and limit of quantification (LOQ) were calculated using the calibration curve method, where σ is the standard deviation of the regression residuals and S is the slope of the calibration curve.

LOD=3.3σ/S

LOQ=10σ/S

##### Repeatability

Six parallel samples of *Panax japonicus* powder were accurately weighed, extracted with methanol, diluted to volume, and analyzed under the chromatographic conditions in Section Chromatographic Conditions to determine the total saponin content. The relative standard deviation (RSD) values were calculated.

$$RSD=\frac{S}{\bar{x}}\times 100\%=\frac{\sqrt{\frac{\sum_{i=1}^{n}{\left(x_{i}-\bar{x}\right)}^{2}}{n-1}}}{\bar{x}}\times 100\%$$

##### Precision

An accurately pipetted 1.0 mL aliquot of the sample solution from Section Preparation of the Test Solution was injected six consecutive times under the chromatographic conditions in Section Chromatographic Conditions. The peak areas of total saponins were recorded, and the RSD was calculated.

##### Stability

An aliquot of the same test solution from Section Preparation of the Test Solution was accurately pipetted and kept at room temperature. The solution was injected at 0, 2, 4, 8, and 12 h under the chromatographic conditions in Section Chromatographic Conditions. The peak areas of total saponins were recorded, and the RSD was calculated.

##### Recovery

Six aliquots of the sample solution with known *Panax japonicus* total saponin content were accurately spiked with appropriate amounts of CS-IVa and G-Ro reference standard solutions from Section Reference Standard Solution Preparation. The resulting solutions were analyzed under the chromatographic conditions described in Section Chromatographic Conditions. The recovery rates and RSD values were calculated based on the peak areas of total saponins.

#### 2.2.4. DES Screening

DES are mainly composed of hydrogen bond donors and hydrogen bond acceptors. These components interact via hydrogen-bond networks, leading to a much lower melting point than that of any single component, allowing DES to exist as liquids at room temperature or upon heating [21]. In the present study, DES were prepared using a heating-stirring method. Individual components were weighed at predetermined molar ratios, mixed in centrifuge tubes, and heated continuously until the mixture became clear and transparent. A suitable amount of deionized water was added and mixed thoroughly before use to obtain the working DES. Details of all tested DES are summarized in Table 1.

DES sample solutions were prepared according to the method in Section Preparation of the Test Solution and injected for determination under the chromatographic conditions described in Section Chromatographic Conditions.

#### 2.2.5. Single-Factor Experiments

##### Molar Ratio

Accurately weighed *Panax japonicus* saponin powder was used to investigate the effects of molar ratios (1:1, 1:2, 1:3, and 2:1) on the saponin content, with the solid-to-liquid ratio, water content, ultrasonic time, and ultrasonic temperature fixed at 1:10, 7%, 40 min, and 60 °C, respectively.

##### Water Content

Owing to the presence of an extensive hydrogen-bonding network, DES generally exhibit considerably high viscosity [22]. Accordingly, an appropriate amount of water was added to reduce the system viscosity and enhance the dispersion of *Panax japonicus* powder in the solvent. In the present study, the effects of water addition (10%, 20%, 30%, 50%, and 60%) on the saponin content were investigated, with all other conditions kept consistent with those in Section Molar Ratio and the molar ratio set at the optimal value determined therein.

##### Solid-to-Liquid Ratio

The effects of solid-to-liquid ratios of 1:10, 1:15, 1:20, 1:25, 1:30, 1:40, and 1:50 on the extracted saponin content were investigated, with all other conditions kept consistent with those in Section Water Content and the water content set at the optimal value determined therein.

##### Ultrasonic Time

The effects of ultrasonic time (30, 40, 50, 60, and 120 min) on the extracted saponin content were investigated, with all other conditions kept consistent with those in Section Solid-to-Liquid Ratio and the solid-to-liquid ratio set at the optimal value determined therein.

##### Ultrasonic Temperature

The effects of ultrasound temperatures at 30, 40, 50, and 60 °C on saponin content were investigated, with all other conditions maintained constant as in Section Ultrasonic Time and the ultrasonic time set at the optimal value obtained therein.

#### 2.2.6. Orthogonal Experiment

Based on the single-factor experiments, the extraction process was systematically investigated by orthogonal array design, using molar ratio (A), solid-to-liquid ratio (B), and water content (C) as factors, and the total content of CS-IVa and G-Ro as the response index (Table 2).

#### 2.2.7. System Setup and MD Simulations

All molecular structures used in MD simulations were obtained from the PubChem database (https://pubchem.ncbi.nlm.nih.gov/ (accessed on 18 May 2026)) and converted to optimized three-dimensional conformations. A solvent system with a molar ratio of choline chloride: urea: water = 1:3:6 was constructed, containing a total of 4500 molecules: 2400 water, 1200 urea, 400 choline chloride, and 100 molecules of either G-Ro or CS-IVa. Choline chloride was dissociated into chloride anions and choline cations to ensure a reasonable spatial distribution. Orthorhombic periodic boundary conditions were applied, and the initial density was set close to 1.0 g·cm^−3^. After generating an amorphous initial configuration, energy minimization was performed for the entire system using the OPLS4 force field. This force field has been validated for choline chloride–urea deep eutectic solvents and accurately reproduces their bulk density and viscosity [23]. A vertical downward gravitational acceleration was applied to simulate the aggregation behavior of solutes in solution. A 100 ps simulation was conducted to ensure adequate system equilibration. The energy integration time step was set to 1.2 fs, the pressure was maintained at 1.01 bar, and the simulation temperature was controlled at 300 K. Simulation trajectories were saved in CMS format for subsequent analysis. Due to computational constraints, only a single 100 ns MD simulation was performed.

Following simulation completion, root-mean-square deviation (RMSD) calculations were performed to evaluate the structural stability of the system. Data collected before the RMSD reached a stable threshold of 2 Å were discarded due to system non-equilibrium. To reveal the molecular extraction mechanism, hydrogen-bonding interactions between solutes and different solvent components were analyzed to identify the dominant interacting species. Radial distribution functions (RDFs) between each solvent and the solute were also computed to characterize solvation behavior and the roles of individual components. Data were plotted using GraphPad Prism (version 10.1.2, GraphPad Software, San Diego, CA, USA), and structural snapshots were visualized using PyMOL (https://pymol.org (accessed on 14 February 2026)).

## 3. Results and Discussion

### 3.1. Determination of Total Saponin Content

**Figure 1:**
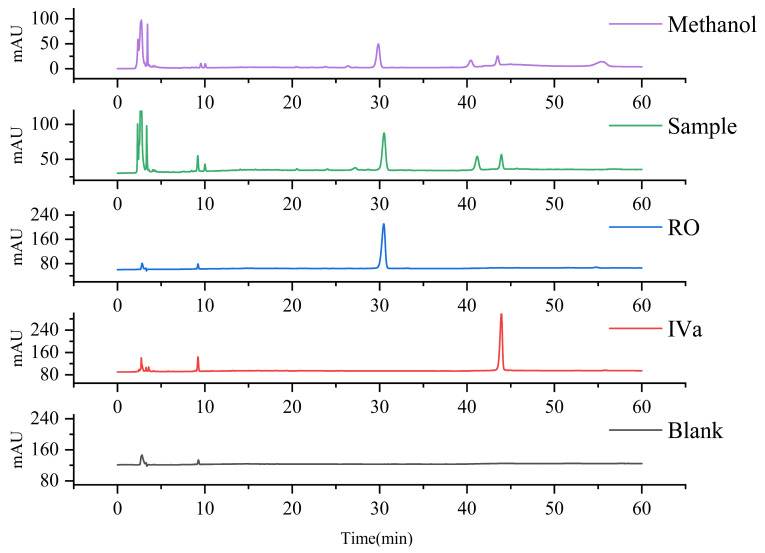
HPLC Chromatograms of blank sample, CS-IVa, G-Ro and methanol extract of *Panax japonicus*.

According to the methods described in the Pharmacopeia of the People’s Republic of China [1,2], the total saponin content in the methanol extract of *Panax japonicus* (calculated as the sum of CS-IVa and G-Ro) was 65.54 mg/g, comprising 11.71 mg/g of CS-IVa and 53.83 mg/g of G-Ro. The corresponding HPLC results for CS-IVa and G-Ro are presented in Figure 1.

### 3.2. Results of Methodological Experiments

#### 3.2.1. Linearity Results

**Figure 2:**
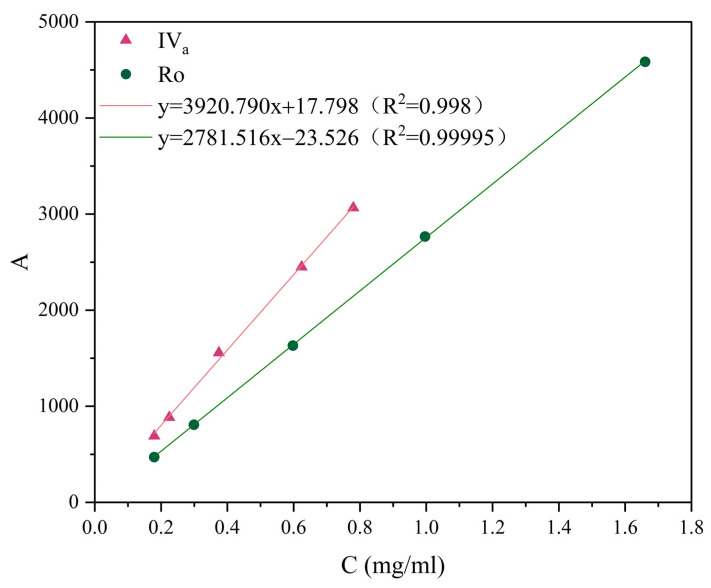
Standard curves of CS-IVa and G-Ro.

The standard curves of CS-IVa and G-Ro are shown in Figure 2. The linear regression equation of CS-IVa was y = 3920.790x + 17.798 (R^2^ = 0.998), indicating good linearity over the concentration range of 0.18–0.78 mg/mL. For G-Ro, the equation was y = 2781.516x − 23.526 (R^2^ = 0.99995), showing good linearity in the range 0.18–1.66 mg/mL.

For Chikusetsusaponin IVa, the calculated LOD was 0.121 mg/mL and the LOQ was 0.367 mg/mL. For Ginsenoside Ro, the LOD was 0.217 mg/mL and the LOQ was 0.658 mg/mL.

#### 3.2.2. Repeatability Results

The RSD for the contents of CS-IVa and G-Ro in the *Panax japonicus* test sample was 1.04% (*n* = 6), as shown in Table 3, demonstrating satisfactory repeatability of the method.

#### 3.2.3. Precision Results

The RSD of the peak area for CS-IVa and G-Ro in the sample solution was 0.33% (*n* = 6), as shown in Table 4, indicating satisfactory instrumental precision.

#### 3.2.4. Stability Results

The RSD of the peak area for CS-IVa and G-Ro in the sample solution was 0.46% (*n* = 5) within 12 h, as shown in Table 5, indicating satisfactory stability of the method.

#### 3.2.5. Recovery Results

The spiked recovery results for CS-IVa and G-Ro are presented in Table 6. The average recovery was 101.852% with an RSD of 1.43% (*n* = 6), indicating that the method is accurate for the determination of CS-IVa and G-Ro contents.

### 3.3. Screening Results of DES

**Figure 3:**
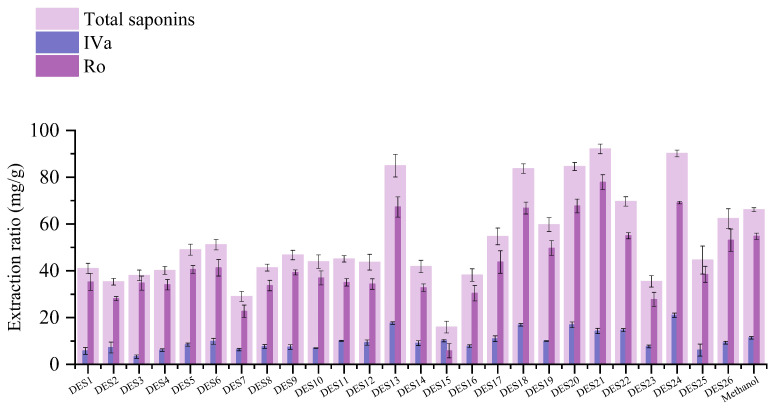
Extraction yields of saponins in various DES.

The extraction performance of different DES was investigated using the total extraction yield (sum of CS-IVa and G-Ro contents) as the response, with results presented in Figure 3.

$$\mathrm{Content}\;\left(\mathrm{mg}/g\right)=\frac{C_{s}\times \frac{A_{\mathrm{sam}}}{A_{s}}\times V}{m_{\mathrm{tot}}}\times 100\%$$

DES21 (betaine–sucrose) showed the best extraction performance, with a total saponin content of 90.68 mg/g, comprising 15.05 mg/g CS-IVa and 75.63 mg/g G-Ro. DES24 (choline chloride–urea) ranked second, with a total saponin content of 89.14 mg/g, including 20.37 mg/g CS-IVa and 68.77 mg/g G-Ro.

At present, the bioactivities of G-Ro have been widely investigated [22,24], whereas studies on CS-IVa remain scarce [25], which may be attributed to the lack of efficient extraction or preparation methods for CS-IVa. Therefore, this study focused on the extraction yield of CS-IVa. Since the extraction performances of DES21 and DES24 were similar, but DES24 provided a higher extraction yield of CS-IVa, DES24 was chosen as the optimal DES for subsequent experiments.

### 3.4. Single-Factor Experiment Results

#### 3.4.1. Molar Ratio Screening Results

**Figure 4:**
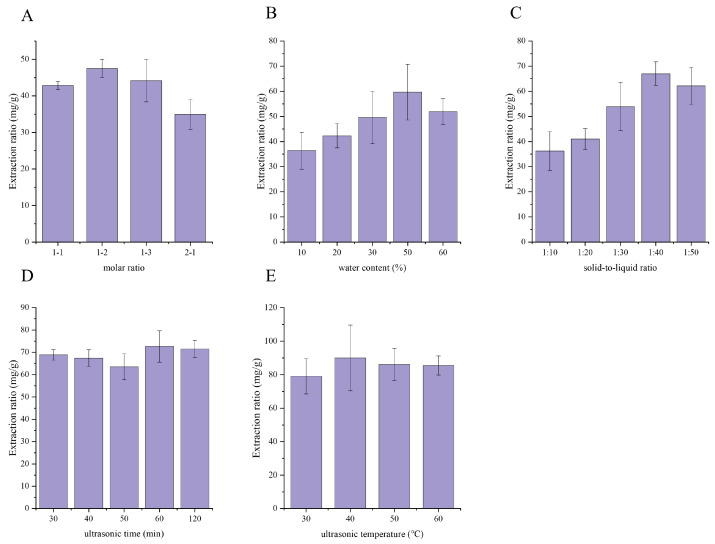
Results of single-factor experiments. Effects of molar ratio (**A**), water content (**B**), solid–liquid ratio (**C**), ultrasonic time (**D**), and ultrasonic temperature (**E**) on the extraction yield of saponins from *Panax japonicus*. Data are presented as mean values of three independent experiments.

As shown in Figure 4A, the total extraction yield of CS-IVa and G-Ro increased gradually with an increasing urea proportion, whereas an excessively high urea content caused a decrease in the extraction yield. This could be attributed to the fact that a moderate amount of urea reduced the viscosity and surface tension of the DES system, thereby enhancing the diffusion and mass transfer efficiency of the target components [26]. The highest total extraction yield was obtained at a choline chloride–urea molar ratio of 1:2, which was thus selected for subsequent single-factor experiments.

The above inferences regarding viscosity and surface tension were primarily derived from literature data, as these properties were not systematically measured in this study for DES with different molar ratios. Consequently, future work should further characterize the physicochemical properties of DES with different molar ratios to establish the structure–activity relationship between these properties and extraction efficiency, thereby providing a more solid theoretical basis for the rational design of DES.

#### 3.4.2. Water Content Screening Results

As shown in Figure 4B, the total extraction yield of CS-IVa and G-Ro first increased and then decreased with increasing water content. A moderate water content reduced the viscosity of the DES system, thus enhancing both the dispersion of *Panax japonicus* powder and mass transfer efficiency. However, an excessively high water content could disrupt the internal hydrogen-bonding interactions of the DES [27], leading to a decrease in extraction efficiency. Accordingly, a water content of 50% was chosen for subsequent single-factor experiments.

#### 3.4.3. Solid–Liquid Ratio Screening Results

As shown in Figure 4C, the total extraction yield of CS-IVa and G-Ro increased with increasing solid–liquid ratio, suggesting that a higher solvent dosage facilitated the sufficient dissolution of saponins. The yield reached a maximum at a solid–liquid ratio of 1:40, and further increasing the solvent volume did not lead to additional improvement. Taking extraction efficiency and solvent cost into comprehensive consideration, a solid–liquid ratio of 1:40 was chosen as the optimal value for subsequent single-factor experiments.

#### 3.4.4. Ultrasonic Time Screening Results

As shown in Figure 4D, the total extraction yield of CS-IVa and G-Ro increased slightly with prolonged ultrasonic time, indicating that proper extension of ultrasonic duration facilitated the gradual dissolution and sufficient release of the target components. The yield reached its maximum at 60 min, and further prolonging the ultrasonic time did not result in significant enhancement of extraction efficiency. Considering both extraction efficiency and energy consumption cost, 60 min was chosen as the optimal ultrasonic time for subsequent single-factor experiments.

#### 3.4.5. Ultrasonic Temperature Screening Results

As shown in Figure 4E, temperature exerted a relatively small effect on the total extraction yield of CS-IVa and G-Ro. Within the experimental range, the yield increased slightly with rising temperature and peaked at 40 °C, whereas further elevation in temperature did not lead to a significant increase. Considering extraction efficiency, energy consumption, and the thermal stability of saponins, 40 °C was chosen as the optimal ultrasonic temperature.

### 3.5. Orthogonal Experiment Results

As shown in Table 7, the influence degree of the three factors on the total extraction yield of CS-IVa and G-Ro followed the order: A (molar ratio) > B (solid–liquid ratio) > C (water content). According to the k_1_, k_2_ and k_3_ values, the optimal level combination for the total saponin yield was A_3_B_3_C_3_. Analysis of variance (Table 8) demonstrated that factor A exhibited a significant effect on the extraction efficiency (*p* < 0.05), while factors B and C showed no significant influence. On the basis of the overall results, the optimal extraction conditions were determined as A_3_B_3_C_3_: molar ratio 1:3, solid–liquid ratio 1:50, and water content 60%, with ultrasonic extraction at 40 °C for 60 min.

#### Process Validation

Three samples of 0.5000 g *Panax japonicus* powder were accurately weighed and extracted in parallel under the aforementioned optimized conditions for process validation. The results for CS-IVa and G-Ro total saponin content are shown in Table 9.

As shown in Table 9, the average total saponin content (sum of CS-IVa and G-Ro) was 74.188 mg/g, with a RSD of 1.18% (≤2%), demonstrating that the optimized extraction process was reproducible, stable and reliable. The contents of individual saponins were 9.59 mg/g for CS-IVa and 64.59 mg/g for G-Ro, respectively.

In the present study, using the extraction yields of CS-IVa and G-Ro as the evaluation indicators, various DES formulations were screened and optimized to obtain the optimal extraction process: a choline chloride to urea molar ratio of 1:3, water content of 60%, solid–liquid ratio of 1:50, ultrasonic time of 60 min, and extraction temperature of 40 °C. Under these conditions, the content of saponins reached 74.188 mg/g, a 13% increase compared with the pharmacopoeial methanol extraction method (65.54 mg/g), indicating significantly superior extraction efficiency over the conventional method.

Notably, the saponin content obtained under the optimized conditions in this study was slightly lower than the maximum value obtained in the orthogonal test. This discrepancy can be rationally explained from two perspectives. First, although the water content in the DES system was appropriately increased, the solvent still exhibited considerable viscosity, which may limit sufficient contact between the solvent and *Panax japonicus* material and reduce mass transfer efficiency. Second, variations in manual mixing during ultrasonic extraction, such as inconsistent mixing intensity and frequency, may also give rise to fluctuations in extraction performance. Nevertheless, the DES-based extraction process developed herein is still significantly superior to the conventional methanol method, fully illustrating that DES hold great potential for the efficient, stable and green extraction of chikusetsusaponins, and can serve as a promising alternative to traditional organic solvents.

### 3.6. MD Simulation Results

**Figure 5:**
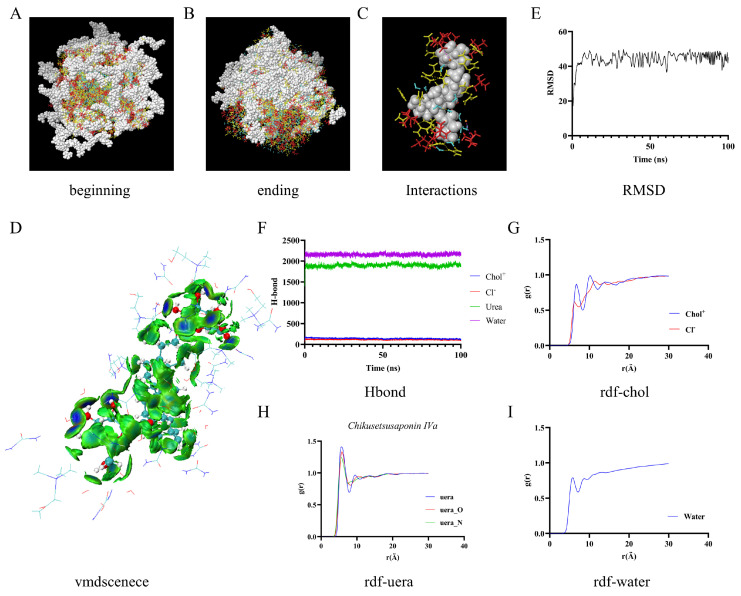
MD simulation results of CS-IVa. (**A**): Initial structure of the simulation system; (**B**): Equilibrated structure of the system after 100 ns simulation; (**C**): Intermolecular interactions between CS-IVa and DES components; (**D**): Visualization of the molecular structure of the simulation system; (**E**): RMSD of the system as a function of simulation time (**F**): Time evolution of the number of hydrogen bonds between CS-IVa and individual DES components; (**G**–**I**): RDFs of CS-IVa with choline chloride, urea, and water. Data were obtained from 100 ns molecular dynamics simulations. In panels (**A**–**D**): white, saponin molecules; red, choline chloride; yellow, urea; cyan, water molecules.

**Figure 6:**
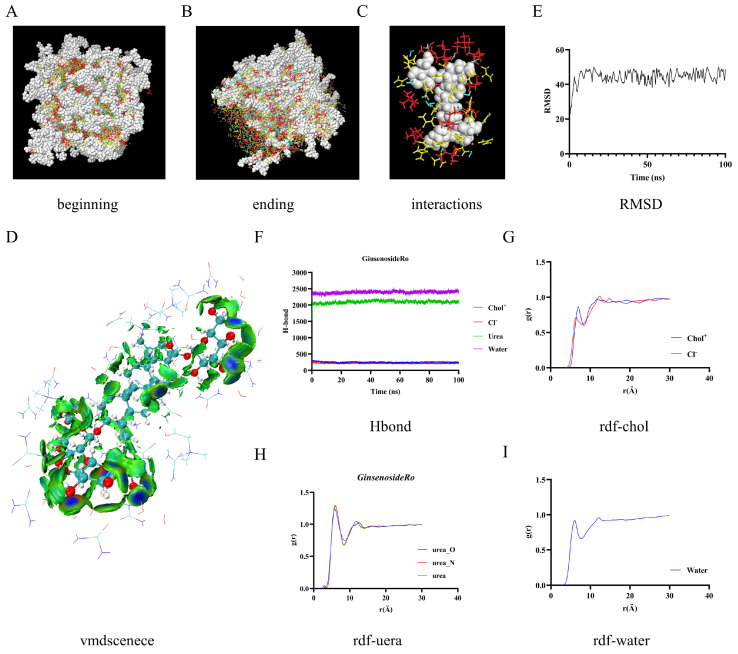
MD simulation results of G-Ro. (**A**): Initial structure of the simulation system; (**B**): Equilibrated structure of the system after 100 ns simulation; (**C**): Intermolecular interactions between G-Ro and DES components; (**D**): Visualization of the molecular structure of the simulation system; (**E**): RMSD of the system as a function of simulation time (**F**): Time evolution of the number of hydrogen bonds between G-Ro and individual DES components; (**G**–**I**): RDFs of G-Ro with choline chloride, urea, and water. Data were obtained from 100 ns molecular dynamics simulations. In panels (**A**–**D**): white, saponin molecules; red, choline chloride; yellow, urea; cyan, water molecules.

To further elucidate the weak intermolecular interactions between DES and chikusetsusaponins, the chikusetsusaponin structure and surrounding DES molecules in the solvated system were extracted. Using Multiwfn software (version 3.8) [28], the modified independent gradient model (mIGM) analysis [29] was performed, and the results were graphically visualized with VMD software (version 1.9.3) [30], as shown in Figure 5 and Figure 6.

#### MD Mechanism of Chikusetsusaponin Extraction by DES

As shown in Figure 5A–I and Figure 6A–I, the solvation processes of the two saponins were highly consistent. At the initial simulation stage (Figure 5A and Figure 6A), saponin molecules were closely aggregated to form a dense core, while DES components were mainly distributed outside the aggregates without sufficient penetration. After 100 ns of simulation (Figure 5B and Figure 6B), the saponin aggregates depolymerized, and urea, choline chloride, and water molecules fully penetrated and surrounded the saponin molecules, forming a stable solvated structure. These results demonstrated that DES could effectively disrupt the intermolecular interactions of saponins, thereby facilitating their efficient dissolution and dispersion. 

Close-up views of intermolecular interactions (Figure 5C and Figure 6C) further revealed the action mode of DES. Urea formed extensive hydrogen bonds with the hydroxyl groups of saponins via its carbonyl oxygen and amino nitrogen, and the hydroxyl group of choline chloride also contributed to hydrogen bonding. These two components competitively occupied the intermolecular hydrogen-bonding sites of saponins, significantly weakening the original intermolecular hydrogen bonds and thus disrupting the aggregation state of saponins.

Spatial distribution function (SDF) maps (Figure 5D and Figure 6D) statistically validated this process. The green isosurface surrounded the hydrophobic core of saponins, corresponding to the hydrophobic effects and van der Waals interactions exerted by choline chloride. The blue isosurface was mainly distributed around the hydroxyl groups of saponins, corresponding to the hydrogen-bonding regions of urea and water, which intuitively reflected the distribution of the solvation shell. The synergistic effect of these two interactions contributed to the effective solvation and disaggregation of saponins.

During the 100 ns molecular dynamics simulations, the RMSD curves of saponins (Figure 5E and Figure 6E) revealed that RMSD values increased rapidly during the initial phase (0–10 ns), indicating that saponin molecules underwent pronounced structural relaxation from the initial conformation to adapt to the choline chloride–urea–water DES system. After 10 ns, RMSD values fluctuated slightly in a stable range, indicating that the system had attained thermodynamic equilibrium and the solvated conformation of saponins remained stable. These results validated the reliability of the simulations and provided a stable structural foundation for the subsequent intermolecular interaction analysis.

Time-dependent curves for the number of hydrogen bonds (Figure 5F and Figure 6F) revealed that water and urea dominated the hydrogen bond network in the system, whereas the contribution of choline cations was relatively small and chloride anions barely formed hydrogen bonds. These findings confirmed that water and urea acted as the key driving force in disrupting the intermolecular hydrogen bond network of saponins. For CS-IVa, water and urea contributed approximately 2200 and 1900 hydrogen bonds at the equilibrium stage, respectively. For G-Ro, the corresponding values were about 2400 and 2100, giving a slightly higher total number of hydrogen bonds than those of the CS-IVa system.

RDF analysis further quantified the distance and strength of intermolecular interactions, with highly consistent interaction profiles observed for both saponin systems. As shown in Figure 5G and Figure 6G, choline cations (Chol^+^) displayed a distinct distribution peak at 5–7 Å, whereas the peak for chloride anions (Cl^−^) was much less intense. These results suggested that Chol^+^ formed strong hydrophobic and van der Waals interactions with the hydrophobic backbone of saponins through its alkyl chain, whereas Cl^−^ only associated with Chol^+^ via electrostatic interactions and did not directly contribute to saponin solvation.

As shown in Figure 5H and Figure 6H, the carbonyl oxygen and amino nitrogen of urea exhibited characteristic hydrogen-bond peaks, with a significantly higher intensity for the carbonyl oxygen, indicating that this group acted as the primary site for hydrogen bonding with saponins. As observed in Figure 5I and Figure 6I, water also displayed distinct hydrogen-bond peaks and provided supplementary stabilization to the hydrogen-bond network. As its main peak intensity was lower than that of urea, these observations indicated a stronger direct interaction between urea and saponins.

Overall, during the 100 ns simulations, Chikusetsusaponin IVa and Ginsenoside Ro exhibited highly consistent solvation behavior. Initially, saponin molecules were tightly aggregated. Over time, urea and water molecules competed for the hydroxyl groups of saponins through extensive hydrogen bonding, disrupting the intermolecular hydrogen bonds between saponin molecules. Meanwhile, choline chloride interacted with the hydrophobic regions of saponins via its alkyl chain through hydrophobic interactions and van der Waals forces, synergistically promoting depolymerization of the saponin aggregates and formation of stable solvated structures. The RMSD curves of the systems reached a plateau after 10 ns, indicating that the simulation systems achieved thermodynamic equilibrium with stable and well-equilibrated conformations. Quantitative analysis revealed that water and urea were the primary contributors to the hydrogen bond network, whereas choline chloride played a weaker role, and chloride ions barely participated in hydrogen bonding. The total number of hydrogen bonds formed by urea and water was slightly higher in the Ginsenoside Ro system (ca. 4500) than in the Chikusetsusaponin IVa system (ca. 4100). This difference was attributed to the greater number of polar hydroxyl groups and sugar chains in the molecular structure of Ginsenoside Ro, which provide more hydrogen-bonding sites, but the core interaction mechanism was identical for both saponins. These findings are consistent with previous reports showing that urea can disrupt the hydrogen bond network in aqueous solutions [31]. Moreover, the contribution of choline chloride to hydrophobic interactions via its alkyl chain aligns with earlier molecular dynamics studies, which demonstrated that the trimethylammonium groups of choline are stabilized by nonpolar interactions at higher concentrations [32], and that the hydrophobic part of choline chloride preferentially faces nonpolar solutes, thereby promoting hydrophobic association [33].

The extraction mechanism of chikusetsusaponins using the choline chloride–urea–water DES is proposed as follows: urea and water molecules compete for the hydroxyl sites of saponins via abundant hydrogen bonds, thus disrupting their intrinsic intermolecular hydrogen-bond network. Choline chloride establishes hydrophobic interactions and van der Waals forces with the hydrophobic core of saponins through its alkyl chain, reducing intermolecular association. The synergistic effect of these two interactions depolymerizes saponin aggregates and stabilizes their dispersion, leading to highly efficient extraction.

Although DES presents operational limitations such as high viscosity and sensitivity to water content, its crystallization behavior has been exploited for the selective purification of polymorphs and phase-change extraction. Moreover, DES can be recovered and is reusable [34]. In addition, ultrasound- or microwave-assisted extraction can significantly reduce mass transfer resistance [35], and viscosity can be lowered by heating or increasing the water content [36]. Alternatively, low-viscosity DES formulations can be developed through the rational design of hydrogen bond donors and acceptors [36]. These strategies provide feasible technical pathways for scaling up DES applications. The hydrogen-bond competition and hydrophobic interaction mechanism revealed in this study provides a molecular basis for optimizing the above strategies and is expected to facilitate the industrial application of DES in the green extraction of saponin-based natural products.

## 4. Conclusions

Chikusetsusaponin extracts exhibit a broad range of pharmacological activities, including anti-inflammatory, hepatoprotective, and cardiovascular protective effects [37,38]. However, efficient extraction methods for chikusetsusaponins remain limited, which hinders the industrial application of these saponins.

In this study, molecular dynamics simulations were employed to elucidate the molecular mechanism of saponin extraction by a choline chloride–urea–water deep eutectic solvent (DES), using Chikusetsusaponin IVa (CS-IVa) and Ginsenoside Ro (G-Ro) as model compounds. The results demonstrate that the DES achieves efficient solubilization and depolymerization of both saponins through a synergistic mechanism involving hydrogen bond competition and hydrophobic interactions. This mechanism was effective for both structurally distinct saponins, suggesting broad applicability. Collectively, this study provides a theoretical basis for the green extraction of saponin-based natural products and offers a methodological reference for molecular simulation studies on DES extraction mechanisms.

## Figures and Tables

**Table 1:** Compositions and molar ratios of DES.

Group	Component	Molar Ratios
DES1	Choline Chloride–Glycerol	1:1
DES2	Choline Chloride–Glycerol	1:2
DES3	Choline Chloride–Ethylene Glycol	1:1
DES4	Choline Chloride–Ethylene Glycol	1:2
DES5	Choline Chloride–Glucose	1:1
DES6	Choline Chloride–Glucose	1:2
DES7	Malic Acid–Sucrose	1:1
DES8	Malic Acid–Sucrose	1:2
DES9	Choline Chloride–Sucrose	1:1
DES10	Choline Chloride–Sucrose	1:2
DES11	Malic Acid–Glucose	1:1
DES12	Malic Acid–Glucose	1:2
DES13	Malic Acid–Glycerol	1:1
DES14	Malic Acid–Glycerol	1:2
DES15	Betaine–Glycerol	1:1
DES16	Betaine–Glycerol	1:2
DES17	Malic Acid–Ethylene Glycol	1:1
DES18	Malic Acid–Ethylene Glycol	1:2
DES19	Betaine–Glucose	1:1
DES20	Betaine–Glucose	1:2
DES21	Betaine–Sucrose	1:1
DES22	Betaine–Sucrose	1:2
DES23	Choline Chloride–Urea	1:1
DES24	Choline Chloride–Urea	1:2
DES25	Betaine–Ethylene Glycol	1:1
DES26	Betaine–Ethylene Glycol	1:2

**Table 2:** Factors and levels of the orthogonal experiment.

Level	A (Molar Ratio)	B (Solid-to-Liquid Ratio)	C (Water Content)
1	1:1	1:30	40%
2	1:2	1:40	50%
3	1:3	1:50	60%

**Table 3:** Repeatability results.

Number	Area
1	1686.8
2	1701
3	1704.9
4	1708.8
5	1662.9
6	1706.6
RSD%	1.04%

**Table 4:** Precision results.

Number	Area
1	1921.8
2	1917.3
3	1921.9
4	1927.5
5	1935.7
6	1924.3
RSD%	0.33%

**Table 5:** Stability results.

Time/h	Area
0	1686.8
2	1699.8
4	1695.1
8	1702
12	1707.5
RSD%	0.46%

**Table 6:** Recovery results.

Number	Sample Content/μg	Added Amount/μg	Found Amount/μg	Recovery %	Average Recovery %	RSD%
1	287.571	635	942.538	103.144%	101.852%	1.43%
2	287.571	635	937.091	102.287%		
3	287.571	317.5	606.822	100.551%		
4	287.571	317.5	608.97	101.228%		
5	287.571	158.75	446.537	100.136%		
6	287.571	158.75	452.295	103.763%		

**Table 7:** Orthogonal experiment results.

Number	A	B	C	Content mg/g
	Molar Ratio	Solid–Liquid Ratio	Water Content %	
1	3	3	1	88.72
2	1	2	3	71.25
3	3	1	3	79.02
4	1	3	2	72.80
5	2	3	3	78.88
6	3	2	2	90.05
7	2	2	1	76.76
8	2	1	2	60.39
9	1	1	1	62.58
K_1_	206.62	201.98	228.06	
K_2_	216.03	238.06	223.23	
K_3_	257.79	240.40	229.14	
k_1_	68.87	67.33	76.02	
k_2_	72.01	79.35	74.41	
k_3_	85.93	80.13	76.38	
R	17.06	12.80	1.97	

**Table 8:** Analysis of variance results.

Source of Variation	Sum of Squares	Degrees of Freedom	Mean Square	F-Value	*p*-Value
A	494.633	2	247.316	22.189	0.043
B	309.187	2	154.593	13.870	0.067
C	6.599	2	3.299	0.296	0.772

**Table 9:** Validation results.

Number	Content (mg/g)	Average Content (mg/g)	RSD%
1	74.065	74.188	1.18%
2	73.558		
3	74.943		

## Data Availability

The original data and contributions presented in this study are available within the article.

## Abbreviations

Deep Eutectic Solvents, DES; Ultrasound-Assisted, UA; Molecular Dynamics, MD; Chikusetsusaponin IVa, CS-IVa; Ginsenoside Ro, G-Ro; Hydrogen Bond Donor, HBD; Hydrogen Bond Acceptor, HBA; Limit of Detection, LOD; Limit of Quantification, LOQ; Relative Standard Deviation, RSD; Root-Mean-Square Deviation, RMSD; Radial Distribution Functions, Rdfs; Modified Independent Gradient Model, Migm; Spatial Distribution Function, SDF.
